# Occupational impacts of early inflammatory arthritis: results from the National Early Inflammatory Arthritis Audit

**DOI:** 10.1093/rheumatology/kead484

**Published:** 2023-09-19

**Authors:** Katie Bechman, Emma S Cook, Edward Alveyn, Abdullah Houssien, Martin Stevens, Mark D Russell, Maryam Adas, Paul Amlani-Hatcher, Sam Norton, Heidi Lempp, Joanna M Ledingham, James B Galloway, Karen Walker-Bone

**Affiliations:** Department of Inflammation Biology, Centre of Rheumatic Disease, King’s College London, London, UK; Department of Inflammation Biology, Centre of Rheumatic Disease, King’s College London, London, UK; Department of Inflammation Biology, Centre of Rheumatic Disease, King’s College London, London, UK; Department of Inflammation Biology, Centre of Rheumatic Disease, King’s College London, London, UK; MRC Lifecourse Epidemiology Centre, University of Southampton, Southampton, UK; MRC Versus Arthritis Centre for Musculoskeletal Health and Work, University of Southampton, Southampton, UK; Department of Inflammation Biology, Centre of Rheumatic Disease, King’s College London, London, UK; Department of Inflammation Biology, Centre of Rheumatic Disease, King’s College London, London, UK; Department of Physiology, Faculty of Medicine, University of Jeddah, Jeddah, Saudi Arabia; NEIAA Patient Panel, British Society for Rheumatology, London, UK; Department of Psychology, Institute of Psychiatry, Psychology and Neuroscience, King’s College London, London, UK; Department of Inflammation Biology, Centre of Rheumatic Disease, King’s College London, London, UK; Rheumatology Department, Portsmouth Hospitals University NHS Trust, Portsmouth, UK; Department of Inflammation Biology, Centre of Rheumatic Disease, King’s College London, London, UK; MRC Versus Arthritis Centre for Musculoskeletal Health and Work, University of Southampton, Southampton, UK; Monash Centre for Occupational and Environmental Health, Monash University, Melbourne, Australia

**Keywords:** inflammatory arthritis, employment, patient-reported outcome measures

## Abstract

**Objectives:**

Inflammatory arthritis causes significant work disability. Studies regarding this frequently fail to report important contextual information such as employment type. Our objective was to explore work participation, by gender and occupation type, in early inflammatory arthritis.

**Methods:**

Data are from the National Early Inflammatory Arthritis Audit for 2018–2020. At diagnosis, clinicians collected information on demographics, inflammatory arthritis disease activity, and working status. Participants completed patient-reported outcomes at baseline, 3 months and 12 months, including occupation and Work Productivity and Activity Impairment (WPAI). Descriptive analyses of work participation and WPAI scores by occupational class at all time points were performed. Regression models were used to examine associations between WPAI score and occupation.

**Results:**

In all, 12 473 people received a diagnosis of inflammatory arthritis and reported employment status, among whom 5999 (47%) were in paid work for at least 20 hours/week. At diagnosis, the working cohort had statistically significant lower measures of disease activity (*P* < 0.001). Occupational data were available for 3694 individuals. At diagnosis, 2793 completed a WPAI; 200 (7.2%) had stopped work and 344 (12.3%) changed jobs because of inflammatory arthritis symptoms. There was a high burden of absenteeism (30%) and presenteeism (40%). Compared with managerial or professional workers, the burden of work disability was greater among those in routine (manual) occupations. During follow-up, 9.4% of WPAI completers stopped work and 14.6% changed roles. Work drop-out occurred almost entirely among people doing routine jobs.

**Conclusion:**

It is easier to retain work in certain employment sectors. Participation in routine jobs is more affected, which may widen health inequalities.

## Graphical Abstract

**Figure:**
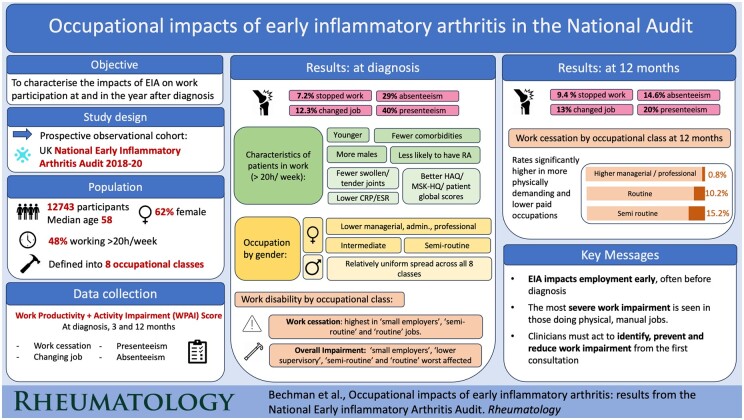

Rheumatology key messages

- Inflammatory arthritis impacts patients’ ability to work, compounding physical and mental health problems.
- Work disability in inflammatory arthritis presents early, often prior to diagnosis, and is more prevalent in routine jobs typically held by poorer-paid individuals.
- Clinicians should address work disability at diagnosis, providing specific advice based on patient employment type.

## Introduction

For most people, working is a very important part of life: it defines status in society; gives purpose and meaning; creates self-esteem; facilitates financial independence; and enables individuals to support dependants [1]. Working is associated with better physical and mental health than worklessness [2]. Taking a societal perspective, maximizing work participation benefits economies directly through gross domestic product and indirectly through reduced welfare payments and increased tax revenue. People with inflammatory arthritis report that remaining in (or returning to) employment is relevant to them [3] and an important goal of treatment [4].

Work non-participation can be broadly measured in three ways: absenteeism, presenteeism and work disability (cessation of work at least partly because of inflammatory arthritis) [5]. Previously, inflammatory arthritis has been shown to cause substantial work disability: early studies suggested that among people in paid employment prior to a diagnosis with RA, 10% became work-disabled within 12 months, rising to 90% after 30 years [6]. After being diagnosed with AS, 50% of patients were eventually unable to work due to their arthritis, when follow up over several decades [7]. The burden of work disability has been found to be high in the first year after diagnosis of RA, which may reflect delays in referral to rheumatologists, diagnosis or treatment [6]. Inflammatory arthritis has been found to cause both absenteeism (work missed per unit time, usually measured in hours, days or weeks of sick leave taken) and presenteeism. Presenteeism is defined as hours of work during which the individual is at work but underperforming because of illness (often measured as a percentage of normal ‘productivity’). Both absenteeism and presenteeism can be difficult to measure [8], and a variety of different outcome measures have been developed, which may be overlapping but are not interchangeable. With improvements in the diagnosis and treatment of inflammatory arthritis, there is evidence that work participation among patients is increasing, although work disability is still more common compared with in the general population [9, 10].

In addition to the measurement challenges, the work participation literature has frequently been criticized for failing to consider relevant contextual factors [11]. Many studies in this field have reported proportions of patients in work *vs* not in work, failing to factor in the number of hours worked, nature of employment, and/or flexibility of working arrangements. There are also inconsistencies regarding the association between gender and work disability [12–14], which in part may relate to differences in work characteristics and demands. A EULAR taskforce therefore developed Points to Consider when measuring work outcomes among patients with rheumatic diseases. It recommended researchers take into account job type and demands, disease-related factors (e.g. inflammation burden, articular symptoms) and comorbidities when designing and reporting work participation outcomes [15]. Data are needed in order to understand the risk factors for poor work outcomes including gender, clinical disease markers and type of employment, and whether successful treatment of inflammatory arthritis improves the ability to work in the short and longer term.

The National Early Inflammatory Arthritis Audit (NEIAA) is a large cohort study commissioned by the Healthcare Quality Improvement Partnership (HQIP). This study aims to investigate the role of clinical factors, diagnosis, and type of employment on work participation cross-sectionally at diagnosis and to describe changes in work participation over the following year, among men and women with early inflammatory arthritis (EIA).

## Methods

### Patient population

The participants were individuals seen in rheumatology services and registered with NEIAA in England and Wales between May 2018 and March 2020. All rheumatology departments were mandated to participate, and data were collected at departmental level. Patients with suspected inflammatory arthritis aged >16 years were eligible for inclusion, but only those with confirmed EIA diagnoses were followed up. The detailed NEIAA methodology has been previously described [16]. For this study, we excluded any individual without a confirmed clinician diagnosis of RA, PsA, AS or undifferentiated arthritis.

### Baseline and longitudinal assessment

At registration into NEIAA, clinicians collected the following patient data: demographic information, ethnicity (observer-assigned), smoking status, number of comorbidities (using the Rheumatic Disease Comorbidity Index list) [17], duration of inflammatory arthritis symptoms and whether in paid work for at least 20 hours/week (yes/no). At baseline and the 3-month and 12-month follow-ups, tender and swollen joint counts, global assessment, ESR and CRP were measured. Data were also collected on whether patients met three key metrics of care based on NICE quality standards [18]: (i) referred within 3 days of presentation, (ii) seen by rheumatology services within 3 weeks of referral and (iii) treatment started within 6 weeks of being seen by rheumatology.

The participants were asked to complete a range of Patient-Reported Outcome Measurements (PROMs) at baseline and at the 3-month and 12-month follow-ups. These included the Musculoskeletal Health Questionnaire (MSK-HQ) [19], the HAQ (HAQ-II) [20] and the Work Productivity and Activity Impairment (WPAI) score [21]. If employed, participants were invited to state their occupation and the industry in which they worked. This information was coded according to the UK Standard Occupational Classification 2010 [22] into the eight National Statistics Socio-Economic Classification (NS-SEC) analytic classes. This validated classification system was constructed in 2001 to measure the employment relations and working conditions derived from the names of occupations to stratify socio-economic circumstances as defined by occupation. The classification is into 8 groups: (i) higher managerial, administrative and professional occupations; (ii) lower managerial, administrative and professional; (iii) intermediate; (iv) small employers and own account workers; (v) lower supervisory and technical; (vi) semi-routine; and (vii) routine (the eighth class is long-term unemployed/never worked, which is not applicable to the current study).

### Outcome measures

Baseline occupational status was defined by whether a patient was in paid work for at least 20 hours/week (chosen to maximize inclusion of part-time workers). Work participation was assessed in all employed participants (including those working <20 hours/week) using the WPAI score [21]. This is in line with recommendations by Leggett and colleagues based on the views of patients from seven countries as to the most useful tool for assessing the impact of disease on work [23]. WPAI was assessed at diagnosis, and at 3 months and 12 months, providing data on the effect of inflammatory arthritis over the previous week on: (i) presenteeism—percentage time that work productivity was affected by inflammatory arthritis symptoms; (ii) absenteeism—hours of work missed as a percentage of total time at work; (iii) overall work impairment—a combination of absenteeism and presenteeism; and (iv) the requirement to change or stop work because of inflammatory arthritis. Absenteeism data were dichotomized into those who reported sick leave and those who did not.

### Statistical analysis

Characteristics of individuals, by employment status and by occupation, were tabulated and compared by gender. The WPAI scores at baseline were calculated and graphically represented in radar plots, by occupation type. The WPAI scores at 3 months and 12 months were also calculated and graphically presented in stacked bar graphs and joy plots.

Regression models were used to examine associations between the eight-class NS-SEC occupation and WPAI score at baseline, with either odds ratios (ORs) from logistic regressions or beta-coefficients (β) from linear regressions reported, depending on the outcome variable from the WPAI score. The ‘higher managerial, administrative, professional’ occupation was used as the reference group for all comparisons. Variables for adjustment were age and sex, decided a priori. Stratified regression analyses by gender were performed to identify any differences between men and women in the associations between occupation category and WPAI score.

WPAI completion relies on patient adherence and is therefore susceptible to a higher rate of missing data compared with clinician-collected information in NEIAA, particularly at the 3-month and 12-month follow-ups. Comparisons between WPAI score completers and non-completers were performed.

### Patient and public involvement

Patients were consulted on the design of the NEIAA from its inception through the NEIAA Patient Panel, with assessment of work status specifically included to facilitate this type of analysis.

### Ethics

Ethical approval for the secondary use of NEIAA data was obtained: REC reference 19/EE/0082. NEIAA has the permission of the Secretary of State for Health to collect data for the purpose of a national audit (Clinical Advisory Group Reference 19/CAG/0059); hence, informed consent was not required for this study.

### Role of funding source

The NEIAA is commissioned by the Healthcare Quality Improvement Partnership (HQIP), funded by NHS England and NHS Improvement and the Welsh government, and is carried out by the BSR, King’s College London, King’s College Hospital and Net Solving. The study sponsors were not involved in the analysis and interpretation of data or the writing of this manuscript.

## Results

### Patients in paid work

In total, 44 410 individuals with suspected inflammatory arthritis were recruited to NEIAA between May 2018 and March 2020, of whom 12 633 received a diagnosis of inflammatory arthritis. Baseline occupational status, defined by whether a patient was in paid work for at least 20 hours/week, was obtained for 12 473 patients. The median age was 58 years [interquartile range (IQR) 46–70], and 62% were female (Table 1).

**Table 1.** Patient demographic and clinical characteristics, and comorbidities, in relation to employment status and gender

	All patients			Male			Female		
	Total	**Employed <20** **h/wk or unemployed**	**Employed >20** **h/wk**	Total males	**Employed <20** **h/wk or unemployed**	**Employed >20** **h/wk**	Total females	**Employed <20** **h/wk or unemployed**	**Employed >20** **h/wk**
	*N* = 12 473	*N* = 6474	*N* = 5999	*N* = 4747 (38%)	*N* = 2308	*N* = 2439	*N* = 7723 (62%)	*N* = 4165	*N* = 3558
**Age (med, IQR)**	58 (46,70)	69 (57,75)	50 (39,57)	61 (49,71)	71 (64,77)	52 (41,59)	56 (44,68)	66 (52,74)	49 (38,56)
**Smoking status**									
Current smoker	2433 (19.5%)	1121 (17.3%)	1312 (21.9%)	1080 (22.8%)	425 (18.4%)	655 (26.9%)	1353 (17.5%)	696 (16.7%)	657 (18.5%)
Ex-smoker	3659 (29.3%)	2147 (33.2%)	1512 (25.2%)	1716 (36.1%)	1019 (44.2%)	697 (28.6%)	1942 (25.1%)	1127 (27.1%)	815 (22.9%)
Never smoked	5592 (44.8%)	2782 (43.0%)	2810 (46.8%)	1679 (35.4%)	723 (31.3%)	956 (39.2%)	3911 (50.6%)	2059 (49.4%)	1852 (52.1%)
**Ethnicity**									
White	10 837 (86.9%)	5662 (87.5%)	5175 (86.3%)	4274 (90.0%)	2124 (92.0%)	2150 (88.2%)	6561 (85.0%)	3537 (84.9%)	3024 (85.0%)
Black British/African/Caribbean	304 (2.4%)	147 (2.3%)	157 (2.6%)	76 (1.6%)	39 (1.7%)	37 (1.5%)	228 (3.0%)	108 (2.6%)	120 (3.4%)
Asian/Asian British	880 (7.1%)	456 (7.0%)	424 (7.1%)	257 (5.4%)	93 (4.0%)	164 (6.7%)	622 (8.1%)	363 (8.7%)	259 (7.3%)
+6 Mixed/Multiple ethnic groups	65 (0.5%)	25 (0.4%)	40 (0.7%)	17 (0.4%)	4 (0.2%)	13 (0.5%)	48 (0.6%)	21 (0.5%)	27 (0.8%)
Other	301 (2.4%)	145 (2.2%)	156 (2.6%)	92 (1.9%)	38 (1.6%)	54 (2.2%)	209 (2.7%)	107 (2.6%)	102 (2.9%)
Not known	86 (0.7%)	39 (0.6%)	47 (0.8%)	31 (0.7%)	10 (0.4%)	21 (0.9%)	55 (0.7%)	29 (0.7%)	26 (0.7%)
**History of Depression**	971 (7.9%)	567 (8.9%)	404 (6.8%)	256 (5.5%)	148 (6.5%)	108 (4.5%)	714 (9.4%)	419 (10.2%)	295 (8.4%)
**One or more Comorbidities** ^a^	5335 (43.3%)	3588 (56.1%)	1747 (29.5%)	2245 (47.8%)	1478 (64.8%)	767 (31.8%)	3087 (40.5%)	2109 (51.3%)	978 (27.8%)
**EIA diagnosis**									
RA	8720 (69.9%)	4908 (75.8%)	3812 (63.5%)	3209 (67.6%)	1763 (76.4%)	1446 (59.3%)	5510 (71.3%)	3145 (75.5%)	2365 (66.5%)
PsA	1565 (12.5%)	541 (8.4%)	1024 (17.1%)	688 (14.5%)	181 (7.8%)	507 (20.8%)	876 (11.3%)	360 (8.6%)	516 (14.5%)
Axial SpA	232 (1.9%)	53 (0.8%)	179 (3.0%)	136 (2.9%)	23 (1.0%)	113 (4.6%)	96 (1.2%)	30 (0.7%)	66 (1.9%)
Undifferentiated arthritis	1956 (15.7%)	972 (15.0%)	984 (16.4%)	714 (15.0%)	341 (14.8%)	373 (15.3%)	1241 (16.1%)	630 (15.1%)	611 (17.2%)
**Symptom duration, months (med, IQR)**	3 (2, 4)	3 (2, 4)	3 (2, 4)	3 (2, 4)	3 (2, 4)	3 (2, 4)	3 (2, 4)	3 (2, 4)	3 (2, 4)
**Tender joint count (med, IQR)**	6 (2, 10)	6 (3, 11)	5 (2, 10)	5 (2, 10)	6 (2, 11)	4 (1, 9)	6 (2, 10)	6 (3, 12)	5 (2, 10)
**Swollen joint count (med, IQR)**	4 (1, 8)	5 (2, 9)	3 (1, 6)	4 (1, 8)	5 (2, 10)	3 (1, 7)	4 (1, 7)	4 (1, 8)	3 (1, 6)
**Global Health Score (med, IQR)**	60 (40, 80)	60 (40, 80)	50.0 (30, 75)	55 (35.0, 78)	60 (40, 80)	50.0 (30, 70)	60 (40, 80)	60 (45, 80)	60 (40, 79)

	All patients			Male			Female		
	Total	**Employed <20** **h/wk or unemployed**	**Employed >20** **h/wk**	Total males	**Employed <20** **h/wk or unemployed**	Total males	**Employed <20** **h/wk or unemployed**	Total males	**Employed <20** **h/wk or unemployed**
	*N* = 12473	*N* = 6474	*N* = 5999	*N* = 4747 (38%)	*N* = 2308	*N *= 4747 (38%)	*N *= 2308	*N* = 4747 (38%)	*N* = 2308
**ESR (mm/h) (med, IQR)**	24 (10, 41)	29 (14, 47)	19 (8, 35)	23 (9, 41)	29 (14, 48)	16 (6, 35)	25 (11, 42)	29 (14, 46)	20 (8, 36)
**CRP (mg/l) (med, IQR)**	10 (4, 27)	13 (5, 32)	8 (3, 21)	13 (5, 33)	17 (6, 40)	10 (4, 27)	9 (4, 23)	12 (4, 29)	6.0 (3, 17)
**HAQ (med, IQR)**	1.0 (0.5, 1.6)	1.2 (0.7, 1.8)	0.9 (0.4, 1.4)	0.9 (0.5, 1.5)	1.1 (0.6, 1.7)	0.8 (0.4, 1.3)	1.1 (0.6, 1.7)	1.2 (0.7, 1.8)	1.0 (0.5, 1.5)
**MSK-HQ Score (med, IQR)**	25 (17, 33)	23 (16, 32)	26 (18, 35)	26 (18, 35)	25 (17, 34)	27 (19, 36)	24 (16, 32)	23 (15, 32)	25 (17, 34)
**Quality Statement (QS)** ^b^									
1 presentation to referral in ≤3 days	5096 (41.3%)	2601 (40.5%)	2495 (42.1%)	1950 (41.5%)	930 (40.7%)	1020 (42.3%)	3144 (41.1%)	1671 (40.5%)	1473 (41.8%)
2 referrals to rheumatology review in ≤3 wks	5429 (43.9%)	2932 (45.6%)	2497 (42.0%)	2109 (44.8%)	1081 (47.2%)	1028 (42.5%)	3319 (43.3%)	1851 (44.7%)	1468 (41.7%)
3 rheumatology reviews to treatment in ≤6 wks	5505 (44.1%)	2929 (45.2%)	2576 (42.9%)	2105 (44.3%)	1077 (46.7%)	1028 (42.1%)	3398 (44.0%)	1852 (44.5%)	1546 (43.5%)

Median and IQR presented, unless otherwise specified.  IQR: interquartile range; MSK-HQ: musculoskeletal health questionnaire; EIA: early inflammatory arthritis; med: median; IQR: interquartile range; h/wk: hours per week.

a Comorbidity = One or more of the following: heart disease, hypertension, lung disease, depression, cancer, ulcer disease, fracture, diabetes.

b QS1: referred within 3 days of presentation. QS2: seen by rheumatology services within 3 weeks of referral. QS3: treatment started within 6 weeks of being seen by rheumatology.

Forty eight percent (5999/12 473) of patients worked >20 hours/week. Compared with those working <20 hours/week or unemployed (6475/12 473), individuals working >20 hours/week were younger [median age 50 (IQR 39–57) *vs* 69 (IQR 57–75) years, *P* ≤ 0.001], more likely to be male (41% *vs* 36%, *P* < 0.001) and had fewer comorbidities (30% reporting at least one comorbidity *vs* 56%, *P* < 0.001). At diagnosis, measures of disease activity, including swollen and tender joint counts, patients’ global assessment scores, and inflammatory markers, were lower among those in paid work; [e.g. median swollen joint counts 3 (IQR 1–6) *vs* 5 (IQR 2–9), *P* < 0.001 and median CRP 8 (IQR 3–21) *vs* 13 (5–32) *P* < 0.001]. Patient-reported outcomes, including HAQ and MSK-HQ scores, were more favourable in the working cohort [median HAQ 0.9 (IQR 0.4–1.4) *vs* 1.2 (0.7–1.8) *P* < 0.001, and median MSK-HQ 26 (IQR 18–35) *vs* 23 (IQR 16–32) *P* < 0.001]. A similar pattern was seen in men and women.

The commonest diagnoses were RA (69.9%, *n* = 8720/12 473), PsA (12.5%, *n* = 1565/12 473), undifferentiated arthritis (15.7%, *n* = 1956/12 473) and axial SpA (AxSpA) (1.8%, *n* = 232/12 473). A greater proportion of patients diagnosed with RA were not in paid work compared with patients with PsA and AxSpA (56% *vs* 35% and 23%, respectively) (Supplementary Tables S1 and S2, available at *Rheumatology* online). The high proportion unemployed with RA was similar when analysed by gender, suggesting that unemployment was not driven by the female predominance of a diagnosis of RA.

### NS-SEC occupational classification

**Figure 1.**
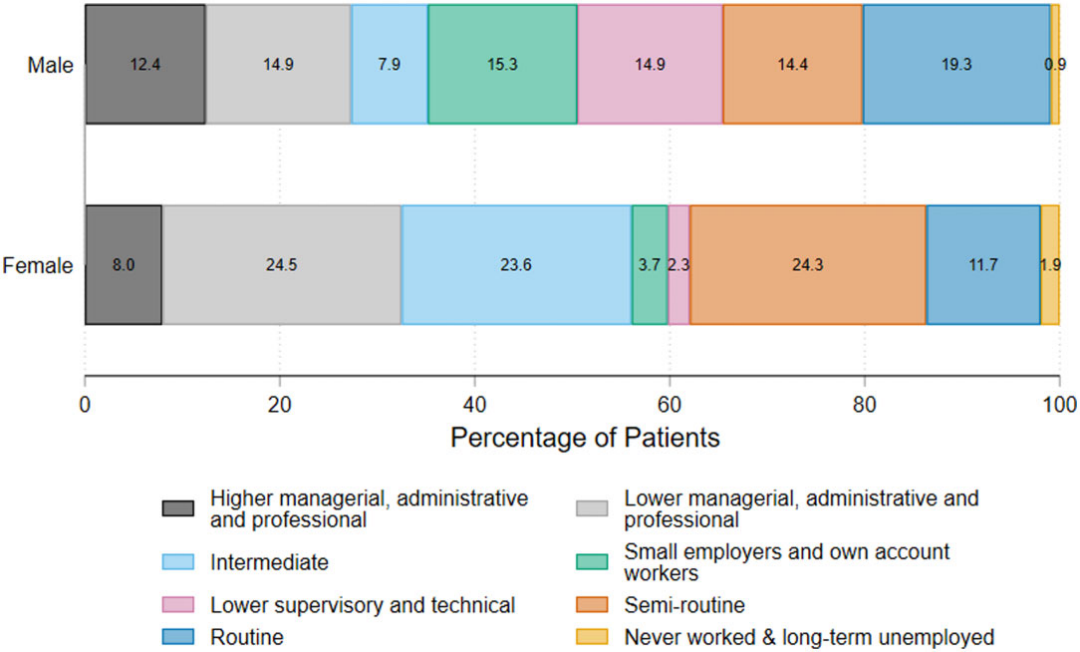
Comparison of occupational classifications of men and women reporting working at time of registration into the National Early Inflammatory Arthritis Audit

Of the 12 473 individuals who provided occupational status, occupational title and industry were available for 3694 individuals (Supplementary Fig. S1, available at *Rheumatology* online). Two-thirds of these individuals were working <20 hours/week. Participants were most commonly in ‘lower managerial, administrative, professional’ (768/3694), ‘intermediate’ (646/3694) and ‘semi routine’ (754/3694) occupational classifications, with a high proportion of females in these three categories. In contrast, occupations of males were spread more evenly across all eight classes (Fig. 1).

Disease activity measures at diagnosis, including tender and swollen joint counts, were statistically similar among the occupational groups. CRP levels were significantly higher and global assessment of health, HAQ and MSK-HQ scored significantly worse among people doing ‘routine’ occupations compared with ‘higher and lower administrative, professional’ workers [median CRP 10 (IQR 4–26) *vs* 7 (IQR 3–22) *P* = 0.02; median global Health 60 (IQR 40–80) *vs* 50 (IQR 30–70) *P* < 0.001; median HAQ 1.1 (IQR 0.6–1.6) *vs* 0.8 (IQR 0.4–1.3) *P* < 0.001; median MSK-HQ 23 (IQR 17–32) *vs* 28 (IQR 20–37) *P* < 0.001] (Supplementary Table S3, available at *Rheumatology* online).

### WPAI score at diagnosis

Of the 12 473 individuals who provided occupational status, 2793 completed a WPAI measure at diagnosis. Compared with patients who did not complete their WPAI measure, these patients were younger, had more comorbidity, presented with less active disease and were more likely to be working >20 hours/week. In fact, over 80% (2274/2793) were working >20 hours/week. Twelve percent (344/2793) reported having changed job due to their inflammatory arthritis symptoms, and 7.2% (200/2793) reported that they had stopped working due to inflammatory arthritis symptoms (Supplementary Tables S1 and S2, available at *Rheumatology* online). Of those still working, 29% of individuals (*n* = 689) reported absenteeism. The median reported presenteeism was 40% (IQR 20–70%). Data on overall work impairment, reported as percentages and representing both absenteeism and presenteeism, were skewed with a wide IQR (median 30%, IQR 5.5–50%)—a consequence of the data spread on absenteeism.

Overall, fewer females had stopped work due to inflammatory arthritis symptoms at diagnosis (5.8% *vs* 9.5% for males). Female patients reported higher rates of presenteeism [50% (20–70%) *vs* 40% (20–70%) for males]. The proportion of patients stopping work or changing roles was similar across the inflammatory arthritis conditions. However, patients with RA reported greater absenteeism and presenteeism than those with other diagnoses [percentage reporting absenteeism in RA 31.4%, PsA 23.8% AxSpA 21.8% and undifferentiated arthritis 27.9% *P* = 0.011] [median presenteeism in RA 50 (IQR 20–70), PsA 30 (IQR 10–60), AxSpA 40 (IQR 10–60) and undifferentiated arthritis 40 (IQR 10–70), *P* < 0.001]. There was no statistically significant difference in overall work impairment reported by people with different conditions.

**Figure 2.**
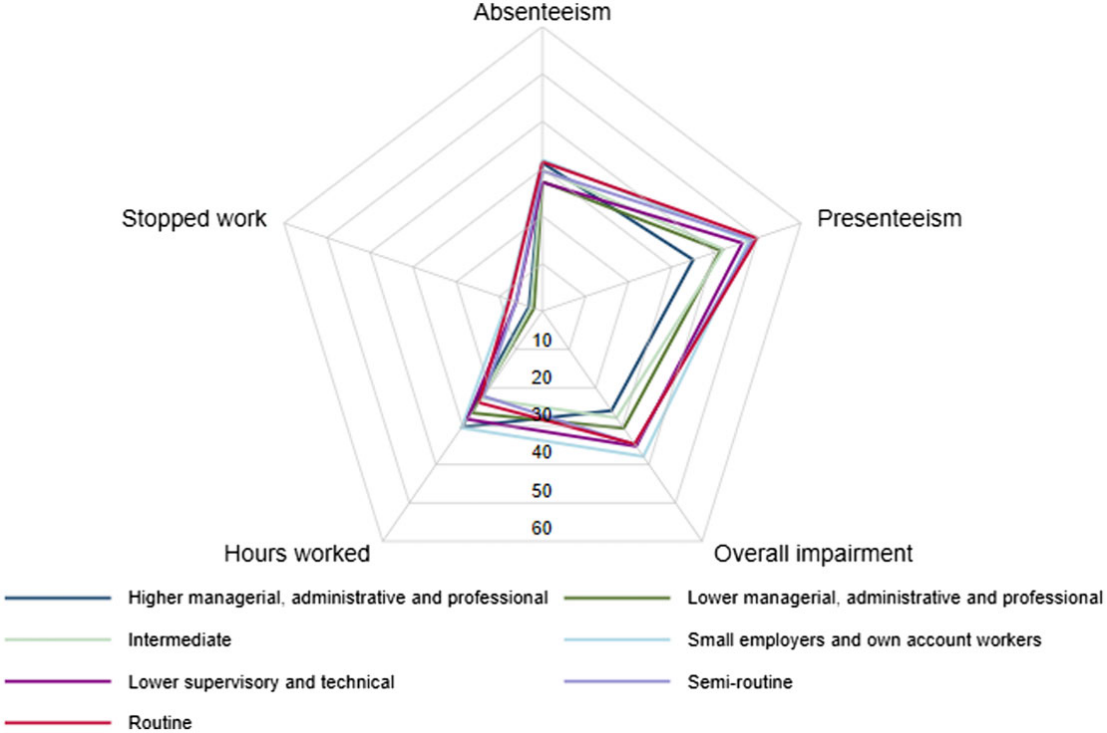
Radar plot of WPAI scores at diagnosis. Stopping work: reported as a percentage of patients who stopped work due to EIA. Absenteeism: reported as a percentage of patients reporting absence due to illness in last 7 days. Presenteeism: reported as a percentage of work productivity. Overall impairment: reported as a percentage, combining levels of absenteeism and presenteeism. Hours worked: reported as hours worked on average each week. WPAI: Work Productivity Activity Impairment; EIA: early inflammatory arthritis

**Figure 3.**
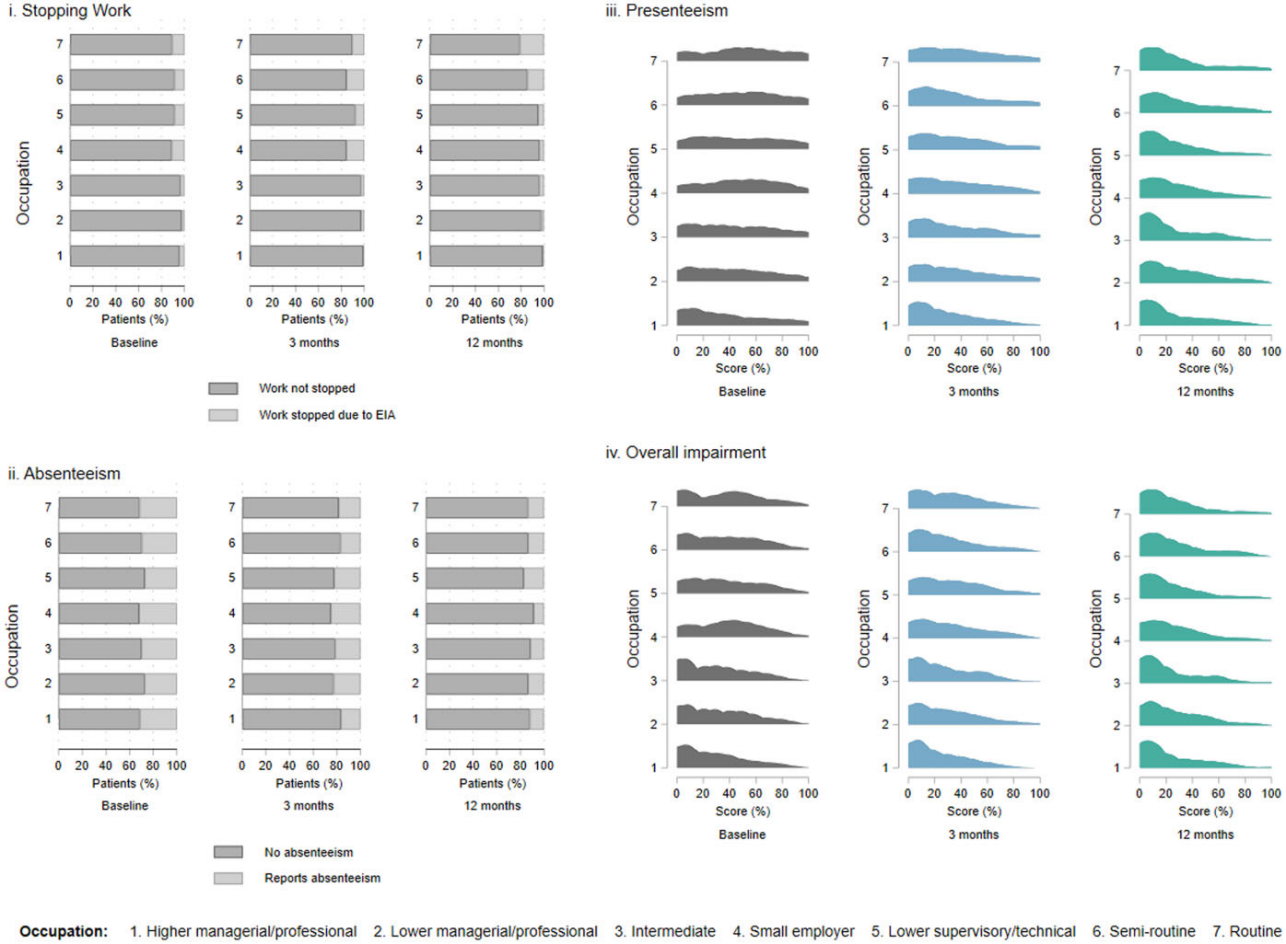
Comparison of WPAI scores. Comparison of WPAI scores for **i.** stopping work due to EIA and **ii.** absenteeism (percentage reporting absence due to illness in last 7 days) **ii.** presenteeism (percentage of work productivity in the last 7 days) and **iv.** overall impairment (percentage combining levels of absenteeism & presenteeism) at diagnosis, 3 month and 12 month time points, by occupational classification. Graphically represented in stacked bar graph for stopping work and absenteeism and joy plots for presenteeism and overall impairment

WPAI scores at diagnosis by occupational classification are graphically presented in a radar plot (Fig. 2 and Supplementary Fig. S2, available at *Rheumatology* online). The percentage of participants stopping working due to inflammatory arthritis symptoms was highest in the ‘small employers/own account works’, ‘lower supervisory/technical’, ‘semi routine’ and ‘routine’ occupations, (ranging from 6% to 8%). The trend of fewer females stopping work at diagnosis was seen in nearly all occupational groups. Comparable rates of absenteeism were seen across the occupational groups. Patients working in occupations classified as ‘small employers/own account works’, semi routine’ and ‘routine’ reported more presenteeism than ‘higher managerial, administrative, professional’ workers. Overall work impairment was lowest in ‘higher managerial, administrative, professional’ occupations (Fig. 3).

### The association between occupation and WPAI score in newly diagnosed inflammatory arthritis

**Figure 4.**
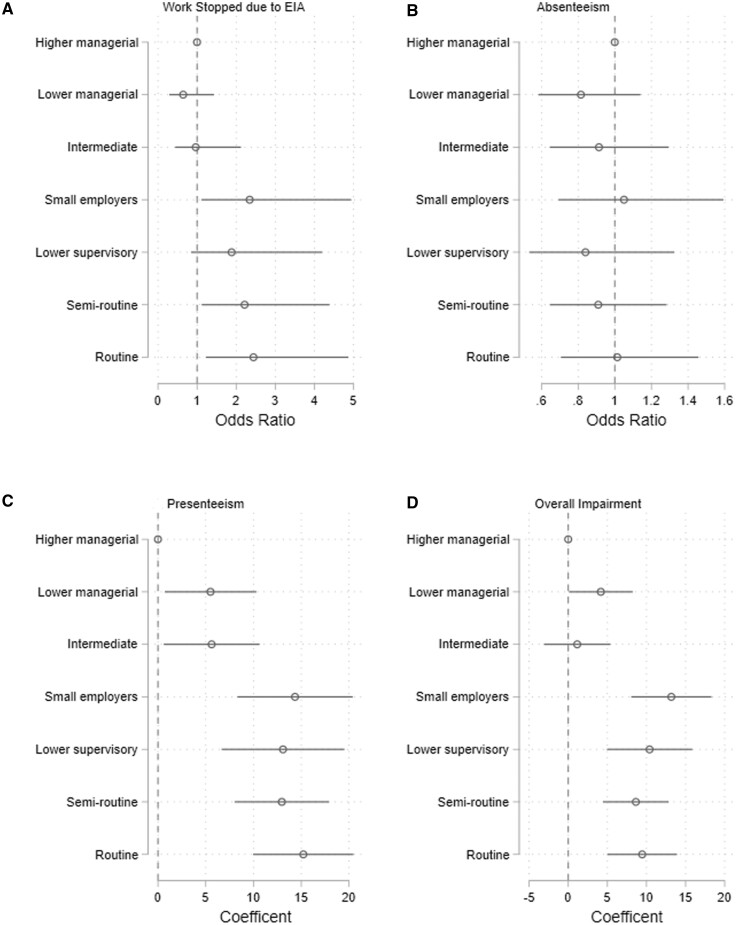
Logistic regression models examining the association between occupational group and a) having stopped working because of inflammatory arthritis b) absenteeism, c) presenteeism, d) overall work impairment at diagnosis. Reference group is ‘higher managerial, administrative, professional’. Adjusted for age and gender

At diagnosis, compared with the ‘higher managerial, administrative, professional’ group, the likelihood of reporting having stopped work was greater among those in occupations classified as ‘small employers/own account works’, ‘semi routine’ and ‘routine’ [adjusted ORs 2.3 (95% CI 1.1–4.9), 2.1 (1.1–4.4) and 2.4 (1.2–4.9), respectively] (Fig. 4). There were no associations between occupational group and reported absenteeism. Compared with ‘higher managerial, administrative, professional’ participants, workers in all other categories reported more presenteeism, with the greatest proportion reported by those in the ‘small employers/own account works’, ‘lower supervisory, technical’, ‘semi routine’ and ‘routine’ groups: beta-coefficient (β) 14.3 (95% CI 8.3–20.4), β 13.1 (6.7–19.5), β 13.0 (8.0–17.9) and β 15.2 (10.0–20.5), respectively. Similar findings were seen for overall work impairment.

Stratified regression analyses by gender demonstrated differences between males and females. Association between occupation category and stopping work was seen among males in the ‘semi-routine’ group. There was no statistically significant association between occupation category and stopping work among females. In contrast, association between occupation category and presenteeism was more pronounced among females than among males. Compared with the ‘higher managerial, administrative, professional’ occupation, female workers in all other categories reported more presenteeism. This association was only seen in male workers from the ‘small employers’, ‘lower supervisory, technical’, ‘semi routine’ and ‘routine’ categories (Supplementary Fig. S3, available at *Rheumatology* online).

### WPAI score longitudinally over 12 months from diagnosis

Fewer individuals completed a WPAI at 3 months (*n* = 1458/12 473, 12%) and 12 months (*n* = 843/12 473, 7%) compared to at diagnosis. WPAI completers at 3 months and 12 months were younger and had less comorbidity, but there was no difference in markers of inflammatory arthritis disease activity, compared with WPAI non-completers. At 12 months, 9.4% of individuals (*n* = 79/843) had stopped work and 14.6% (*n* = 123/843) had changed roles due to their inflammatory arthritis. The proportions who reported stopping work by 12 months were highest in the ‘semi routine’ and ‘routine’ occupational groups. Overall, there were improvements in the proportion of individuals reporting absenteeism (19.4% at 3 months and 13.0% at 12 months) and presenteeism [30% (IQR 10–50) at 3 months and 20% (IQR 0–40) at 12 months] (Fig. 3).

## Discussion

This analysis describes the occupational status of patients at the time of presentation with inflammatory arthritis and during 12 months of follow-up. Despite developing standards for rapid referral after symptom onset [18] and a median duration of symptoms of 3 months, at diagnosis 19% of patients had stopped working or changed jobs due to inflammatory arthritis, while 30% reported having to take time off work due to inflammatory arthritis. Unsurprisingly, those managing paid work had less severe clinical features and less functional impact from their inflammatory arthritis. Work disability prior to diagnosis has not been reported previously in UK cohorts. The proportion in NEIAA stopping work after diagnosis was similar to that reported in the early RA Network cohort (ERAN) published 10 years ago [24], but lower than that in the Norfolk Arthritis Register (NOAR) 20 years ago [25].

Differences by occupational classification were apparent: compared with individuals working as higher managers, administrators or professionals, people working in small businesses or on their own accounts, lower supervisory, technical, semi-routine and routine occupations were more likely to have stopped work at baseline, and to report presenteeism and overall work impairment, but not absenteeism. Rates of work cessation due to inflammatory arthritis remained higher in these same occupational groups over 12 months of follow-up, compared with the ‘higher managers, administrative or professionals’ group. In general, presenteeism and overall work impairment improved longitudinally.

Our finding that work impacts are greater among certain types of workers is not surprising. ‘Manual’ or ‘blue collar’ employment has been found to be associated with work disability in the past [6]. Clearly, if a person’s job requires heavy lifting, performance of manual tasks requiring gripping or pressure, kneeling/squatting or prolonged sitting, with some types of inflammatory arthritis that person will experience more difficulty than someone doing a different job. These types of tasks are more commonly required among the adversely impacted occupational groups identified in our analyses. It is important for rheumatologists to have this specific knowledge if they are to provide relevant career advice. Patients emphasize the importance of work [26], so signposting by clinicians and/or occupational therapists (OTs) for support and retraining will be essential, given that within 2–3 years of diagnosis certain jobs may be impossible for many people with inflammatory arthritis. We recommend that the rheumatology team asks patients with early inflammatory arthritis about their work and provides support for work among patients who request this. OTs have particular strength in this area, and trial data has demonstrated that OT intervention is associated with improved work-related outcomes [27, 28]. Early intervention is crucial for those with rheumatic diseases, as prolonged sickness absence is already recognized to reduce the chances of ever working again, particularly once an individual has not worked for longer than 6–12 months [1, 29]. The economic and health consequences of unemployment, as well as the stigma associated with needing to seek welfare benefits, are all good reasons to prevent job loss where possible. Importantly, our analyses demonstrate that the burden on work ability is being felt most among those at greatest socio-economic disadvantage. NEIAA data have already shown that individuals living in more deprived areas have higher levels of disease activity longitudinally [16], and the current analyses suggest that work disability will further compound this deprivation: those who do the poorest-paid, most physically demanding work, which requires the best possible disease control, are those least likely to achieve it. Unemployment due to illness will only serve to widen inequality, placing a greater burden on the individuals least able to bear it.

That absenteeism is the only work outcome that does not show differences by occupational classification is unsurprising. Absenteeism is a marker of many different factors: personal, social and work-related [30]. Not everyone is eligible for paid sickness absence, and people in routine or manual types of work are often those who are not. The culture in some workplaces may be less supportive of absenteeism. In some settings (e.g. health and social care), workers are more reluctant to take sick leave for fear of leaving their work undone or delegated to overworked colleagues. Finally, when an individual decides whether or not their symptoms are sufficiently severe to need to take time away from their work, they take a range of factors into account (dependants, caring responsibilities, availability of support from partners, finances, co-workers); therefore, even with similar symptoms, they might reach a different decision [31].

There were subtle differences in work disability between male and female patients; females were less likely to stop working, but reported a greater impact of inflammatory arthritis symptoms on work productivity. This was further illustrated in our stratified regression analyses. We have demonstrated that women with inflammatory arthritis work in different occupations compared with men with the condition. Women are also more likely to be in part-time than full-time roles. Inconsistencies in the literature on work disability by gender may be in part explained by differences in occupation type and demands. Our gender-stratified analyses will enable provision of better information about what advice should be given to working patients with new diagnoses of inflammatory arthritis, and understanding of who is likely to need more support to remain in work.

### Strengths and limitations

The strength of the NEIAA cohort is both its size and widespread geographic completeness. This creates reasonable confidence that this is truly a representative sample of patients diagnosed with early inflammatory arthritis across England and Wales.

However, our results need to be considered alongside some limitations. First, we had near-complete data at baseline for occupational status (only 190 missing entries), but fewer patients completed the PROMs than we had hoped, and attrition rates were also high at the 3-month and particularly 12-month follow-ups. Analyses of NEIAA data have demonstrated an effect of gender on PROM completion, with males being less likely to complete a PROM [32]. If anything, we might expect that participation may be better among patients with greater educational attainment and access to and familiarity with information technology. The results might therefore underestimate the burden of work disability overall, and in particular among workers in manual and routine types of jobs. The WPAI has the benefit of being found the most acceptable tool available by European patients with rheumatic diseases [23], but only reflects absenteeism, presenteeism and overall work impairment during the preceding 7 days. Data are self-reported and, although people have been shown to be accurate at recalling hours or days of sick leave in the past 7 days, assessment of percentage productivity loss is much more subjective and variable. Moreover, the productivity is easier to estimate in some types of occupation (e.g. in manufacturing) than others (e.g. orchestra conductor). There has been a call for better, more objective work outcome measures and international consensus as to which of these to use so that pooling of data across studies becomes easier [15, 33]. Finally, the occupation and industry were coded using NS-SEC, which was a system developed to classify socio-economic status in populations. Consequently, people classified similarly may actually carry out very different jobs. Broadly, people in the first three categories are better paid, more likely to be desk-based and supervising or managing organizations, or are professionals. Broadly, people in the first three categories are better paid, more likely to be desk-based and supervising or managing organizations, or are professionals. Those in the last two categories tend to do more physically demanding work and be paid less, and may be required to work in shifts with less autonomy. However, these are generalizations and the ideal way to know which types of jobs are most difficult to do with different types of inflammatory arthritis would be to collect data about the physical and psychological demands of each person’s employment.

## Conclusion

In summary, the impacts of EIA on work are measurable very early and are unequal. Ability to work is improved when symptoms and disease activity are well controlled. Overall, work impairment and presenteeism reduce over the first 12 months after diagnosis. People with more routine (manual) occupations are more likely to stop working or change their job because of their diagnosis in the first year and have more difficulties with presenteeism and overall work impairment.

## Supplementary Material

kead484_Supplementary_Data

## Data Availability

Data used in this study were collected for the National Early Inflammatory Arthritis Audit and are available on request to the data controllers [the Healthcare Quality Improvement Partnership (HQIP)]. Data are available upon reasonable request by any qualified researchers who engage in rigorous, independent scientific research, and will be provided following review and approval of a research proposal and Statistical Analysis Plan (SAP) and execution of a Data Sharing Agreement (DSA). All data relevant to the study are included in the article. All figures and tables included in this article are original.
