# Supplementary material for: Occupational impacts of early inflammatory arthritis: results from the National Early Inflammatory Arthritis Audit
Source: Rheumatology (Oxford). 2023 Sep 19;63(7):1856–67. doi: 10.1093/rheumatology/kead484 (PMC11215985; doi:10.1093/rheumatology/kead484)

**Supplementary Table S1.** Comparison of clinical characteristics and work participation by diagnosis

|  | **Total** | **Rheumatoid Arthritis** | **Psoriatic Arthritis** | **Axial Spondyloarthritis** | **Undifferentiated Arthritis** |
| --- | --- | --- | --- | --- | --- |
|  | N=12,473 | N=8,720 | N=1,565 | N=232 | N=1,956 |
| **Age (median, IQR)** | 58 (46,70) | 61 (50,71) | 48 (36,58) | 37 (30,49) | 55 (41,68) |
| **Gender** |  |  |  |  |  |
| **Female** | 7,723 (61.9%) | 5,510 (63.2%) | 876 (56.0%) | 96 (41.4%) | 1,241 (63.4%) |
| **History of depression** | 971 (7.9%) | 629 (7.3%) | 163 (10.5%) | 21 (9.3%) | 158 (8.3%) |
| **One or More Comorbidity** | 6,992 (56.7%) | 4,695 (54.3%) | 1,014 (65.5%) | 152 (67.3%) | 1,131 (59.1%) |
| **Symptom Duration, months** | 3.0 (2.0,4.0) | 3.0 (2.0,4.0) | 4.0 (2.0,5.0) | 5.0 (3.0,5.5) | 3.0 (2.0,4.0) |
| **Tender Joint Counts** | 6.0 (2.0,10.0) | 6.0 (3.0,11.0) | 4.0 (2.0,8.0) | 0.0 (0.0,2.0) | 3.0 (1.0,8.0) |
| **Swollen Joint Counts** | 4.0 (1.0,8.0) | 5.0 (2.0,9.0) | 2.0 (1.0,5.0) | 0.0 (0.0,1.0) | 2.0 (0.0,4.0) |
| **Global Health Score** | 60.0 (40.0,80.0) | 60.0 (40.0,80.0) | 50.0 (30.0,70.0) | 50.0 (9.0,70.0) | 50.0 (30.0,70.0) |
| **MSKHQ Score** | 25.0 (17.0,33.0) | 24.0 (16.0,33.0) | 26.0 (18.0,35.0) | 24.5 (16.5,33.0) | 26.0 (18.0,34.5) |
| **Quality Statement (QS) met** |  |  |  |  |  |
| 1 presentation to referred <= 3 day | 5,096 (41.3%) | 3,493 (40.4%) | 688 (44.6%) | 86 (37.7%) | 829 (42.7%) |
| 2 referral to rheum. review <=3 wks. | 5,429 (43.9%) | 3,921 (45.3%) | 593 (38.5%) | 78 (33.6%) | 837 (43.2%) |
| 3 rheum. review to treatment <=6 wks. | 5,505 (44.1%) | 4,446 (51.0%) | 547 (35.0%) | 22 (9.5%) | 490 (25.1%) |
| **Patient in paid work >20h/week** | 5,999 (48.1%) | 3,812 (43.7%) | 1,024 (65.4%) | 179 (77.2%) | 984 (50.3%) |
| **Completed baseline WPAI form** | 2,793 (22.4%) | 1,811 (20.8%) | 434 (27.7%) | 93 (40.1%) | 455 (23.3%) |
| - **Stopped work due to EIA** | 200 (7.2%) | 142 (7.8%) | 27 (6.2%) | 6 (6.5%) | 25 (5.5%) |
| - **Changed job due to EIA** | 344 (12.3%) | 229 (12.6%) | 48 (11.1%) | 11 (11.8%) | 56 (12.3%) |
| - **Reported absenteeism** | 689 (29.3%) | 478 (31.4%) | 87 (23.8%) | 17 (21.8%) | 107 (27.9%) |
| - **Presenteeism** | 40.0 (20.0,70.0) | 50.0 (20.0,70.0) | 30.0 (10.0,60.0) | 40.0 (20.0,60.0) | 40.0 (10.0,70.0) |
| - **Overall Impairment** | 30.0 (5.5,50.0) | 30.0 (8.0,50.0) | 30.0 (9.4,50.0) | 30.0 (10.0,52.5) | 22.4 (1.0,50.0) |
| - **Hours Worked (last 7 days)** | 30.0 (7.0,40.0) | 28.0 (6.0,40.0) | 32.0 (14.0,40.0) | 37.0 (14.0,40.0) | 27.0 (3.0,38.0) |

**Abbreviations**: **IQR** = interquartile range; **MSK**-**HQ** = musculoskeletal health questionnaire; **EIA** = early inflammatory arthritis.

* Comorbidity = 1 or more of the following: heart disease, hypertension, lung disease, depression, cancer, ulcer disease, fracture, diabetes.

** QS1: referred within 3 days of presentation. QS2: seen by rheumatology services within 3 weeks of referral. QS3: treatment started within 6 weeks of being seen by rheumatology.

Data included are for all patients, irrespective of whether occupation status was known. Median, IQR unless otherwise specified.

**Supplementary Table S2.** Comparison of clinical characteristics and work participation by diagnosis and gender

|  | **Total** | | **Rheumatoid Arthritis** | | **Psoriatic Arthritis** | | **Axial Spondyloarthritis** | | **Undifferentiated Arthritis** | |
| --- | --- | --- | --- | --- | --- | --- | --- | --- | --- | --- |
|  | ***Male*** | ***Female*** | ***Male*** | ***Female*** | ***Male*** | ***Female*** | ***Male*** | ***Female*** | ***Male*** | ***Female*** |
|  | N=4,747 | N=7,723 | N=3,209 | N=5,510 | N=688 | N=876 | N=136 | N=96 | N=714 | N=1,241 |
| **Age (median, IQR)** | 61 (49,71) | 56 (44,68) | 64 (54,73) | 58 (47,70) | 47 (35,59) | 48 (36,58) | 35 (29,47) | 41 (32,51) | 60 (45,72) | 53 (39,65) |
| **History of depression** | 256 (5.5%) | 714 (9.4%) | 170 (5.3%) | 458 (8.4%) | 43 (6.3%) | 120 (13.9%) | 12 (9.1%) | 9 (9.6%) | 31 (4.4%) | 127 (10.5%) |
| **One or More Comorbidity** | 2,448 (52.2%) | 4,544 (59.5%) | 1,512 (47.5%) | 3,183 (58.3%) | 467 (68.7%) | 547 (63.2%) | 91 (68.9%) | 61 (64.9%) | 378 (53.9%) | 753 (62.1%) |
| **Symptom Duration, months** | 3.0 (2.0,4.0) | 3.0 (2.0,4.0) | 3.0 (2.0,4.0) | 3.0 (2.0,4.0) | 4.0 (2.0,5.0) | 3.0 (2.0,5.0) | 5.0 (4.0,6.0) | 4.0 (3.0,5.0) | 3.0 (2.0,4.0) | 3.0 (2.0,4.0) |
| **Tender Joint Counts** | 5.0 (2.0,10.0) | 6.0 (2.0,10.0) | 6.0 (2.0,11.0) | 6.0 (3.0,12.0) | 4.0 (2.0,7.0) | 4.0 (2.0,8.0) | 0.0 (0.0,1.0) | 1.0 (0.0,4.0) | 3.0 (1.0,8.0) | 3.0 (1.0,7.0) |
| **Swollen Joint Counts** | 4.0 (1.0,8.0) | 4.0 (1.0,7.0) | 5.0 (2.0,10.0) | 4.0 (2.0,8.0) | 2.0 (1.0,5.0) | 2.0 (1.0,5.0) | 0.0 (0.0,0.0) | 0.0 (0.0,1.0) | 2.0 (0.0,5.0) | 1.0 (0.0,4.0) |
| **Global Health Score** | 55.0 (35.0,78.0) | 60.0 (40.0,80.0) | 60.0 (40.0,80.0) | 60.0 (45.0,80.0) | 50.0 (25.0,70.0) | 55.0 (40.0,70.0) | 50.0 (4.0,70.0) | 50.0 (10.0,72.5) | 50.0 (25.0,70.0) | 50.0 (30.0,70.0) |
| **MSKHQ Score** | 26.0 (18.0,35.0) | 24.0 (16.0,32.0) | 25.0 (17.0,34.0) | 23.5 (16.0,32.0) | 30.0 (21.0,38.0) | 24.0 (16.0,31.0) | 25.0 (16.0,33.0) | 24.0 (17.0,34.0) | 27.0 (18.5,36.0) | 25.0 (17.0,34.0) |
| **Quality Statement (QS) met** |  |  |  |  |  |  |  |  |  |  |
| 1 presentation to referred <= 3 day | 1,950 (41.5%) | 3,144 (41.1%) | 1,292 (40.7%) | 2,200 (40.3%) | 307 (45.5%) | 380 (43.8%) | 45 (33.8%) | 41 (43.2%) | 306 (43.2%) | 523 (42.5%) |
| 2 referral to rheum. review <=3 wks. | 2,109 (44.8%) | 3,319 (43.3%) | 1,464 (45.9%) | 2,457 (44.9%) | 272 (40.2%) | 320 (37.0%) | 42 (30.9%) | 36 (37.5%) | 331 (46.8%) | 506 (41.1%) |
| 3 rheum. review to treatment <=6 wks. | 2,105 (44.3%) | 3,398 (44.0%) | 1,673 (52.1%) | 2,772 (50.3%) | 249 (36.2%) | 297 (33.9%) | 8 (5.9%) | 14 (14.6%) | 175 (24.5%) | 315 (25.4%) |
| **Patient in paid work >20h/week** | 2,439 (51.4%) | 3,558 (46.1%) | 1,446 (45.1%) | 2,365 (42.9%) | 507 (73.7%) | 516 (58.9%) | 113 (83.1%) | 66 (68.8%) | 373 (52.2%) | 611 (49.2%) |
| **Completed baseline WPAI form** | 1,055 (22.2%) | 1,738 (22.5%) | 644 (20.1%) | 1,167 (21.2%) | 196 (28.5%) | 238 (27.2%) | 59 (43.4%) | 34 (35.4%) | 156 (21.8%) | 299 (24.1% |
| - **Stopped work due to EIA** | 100 (9.5%) | 100 (5.8%) | 70 (10.9%) | 72 (6.2%) | 13 (6.6%) | 14 (5.9%) | 3 (5.1%) | 3 (8.8%) | 14 (9.0%) | 11 (3.7%) |
| - **Changed job due to EIA** | 122 (11.6%) | 222 (12.8%) | 79 (12.3%) | 150 (12.9%) | 18 (9.2%) | 30 (12.6%) | 8 (13.6%) | 3 (8.8%) | 17 (10.9%) | 39 (13.0%) |
| - **Reported absenteeism** | 443 (29.8%) | 170 (32.8%) | 308 (30.7%) | 35 (20.6%) | 52 (26.5%) | 10 (19.6%) | 7 (25.9%) | 31 (25.0%) | 76 (29.2%) | 443 (29.8%) |
| - **Presenteeism** | 40.0 (20.0,70.0) | 50.0 (20.0,70.0) | 45.0 (20.0,70.0) | 50.0 (20.0,70.0) | 30.0 (10.0,50.0) | 50.0 (20.0,70.0) | 40.0 (30.0,70.0) | 30.0 (0.0,60.0) | 30.0 (10.0,60.0) | 50.0 (10.0,70.0) |
| - **Overall** **Impairment** | 30.0 (10.0,50.0) | 30.0 (1.0,50.0) | 30.0 (10.0,50.0) | 30.0 (6.8,50.0) | 20.0 (8.2,40.5) | 30.0 (9.7,51.7) | 40.0 (20.0,54.6) | 20.0 (0.1,52.5) | 20.0 (10.0,48.9) | 30.0 (1.0,55.0) |
| - **Hours Worked (last 7 day)** | 37.0 (16.0,41.5) | 24.0 (3.0,37.0) | 35.0 (10.0,40.0) | 24.0 (3.0,37.0) | 40.0 (25.0,45.0) | 24.5 (2.0,37.0) | 40.0 (10.0,42.0) | 28.0 (15.0,40.0) | 37.0 (16.0,42.0) | 22.0 (0.0,37.0) |

**Abbreviations**: **IQR** = interquartile range; **MSK**-**HQ** = musculoskeletal health questionnaire; **EIA** = early inflammatory arthritis.

* Comorbidity = 1 or more of the following: heart disease, hypertension, lung disease, depression, cancer, ulcer disease, fracture, diabetes.

** QS1: referred within 3 days of presentation. QS2: seen by rheumatology services within 3 weeks of referral. QS3: treatment started within 6 weeks of being seen by rheumatology.

Data included are for all patients, irrespective of whether occupation status was known.

Median, IQR unless otherwise specified.

**Supplementary Figure S1.** Population Flow Chart

**Supplementary Table S3.** Comparison of clinical characteristics, patient-reported outcome measures and work participation by occupational group

|  | **Total** | **Higher managerial, administrative, professional** | **Lower managerial, administrative, professional** | **Intermediate** | **Small employers, own account workers** | **Lower supervisory, technical** | **Semi-routine** | **Routine** | **Never worked, long-term unemployed** |
| --- | --- | --- | --- | --- | --- | --- | --- | --- | --- |
|  | N=3,694 | N=358 | N=768 | N=646 | N=304 | N=265 | N=754 | N=542 | N=57 |
| Tender Joint Count | 5.0 (2.0,10.0) | 5.0 (2.0,9.0) | 5.0 (2.0,9.0) | 5.0 (2.0,11.0) | 6.0 (2.0,11.0) | 5.0 (2.0,9.5) | 6.0 (3.0,11.0) | 5.0 (2.0,10.0) | 6.0 (2.0,11.0) |
| Swollen Joint Count | 4.0 (1.0,7.0) | 4.0 (1.0,7.0) | 3.0 (1.0,6.0) | 3.0 (1.0,8.0) | 5.0 (1.0,9.0) | 3.0 (1.0,8.0) | 4.0 (1.0,8.0) | 4.0 (1.0,7.0) | 4.0 (1.0,6.0) |
| Global Health | 60.0 (40.0,80.0) | 50.0 (30.0,70.0) | 50.0 (30.0,70.0) | 60.0 (40.0,80.0) | 60.0 (40.0,80.0) | 50.0 (32.0,79.0) | 60.0 (40.0,80.0) | 60.0 (40.0,80.0) | 70.0 (50.0,80.0) |
| HAQ score | 1.0 (0.5,1.5) | 0.8 (0.4,1.3) | 0.9 (0.5,1.5) | 1.0 (0.5,1.6) | 1.0 (0.6,1.5) | 0.9 (0.4,1.4) | 1.1 (0.6,1.6) | 1.1 (0.6,1.6) | 1.5 (1.1,2.0) |
| MSK-HQ score | 25.0 (17.0,34.0) | 28.0 (20.0,37.0) | 26.0 (18.0,34.0) | 25.0 (18.0,34.0) | 25.0 (17.5,33.0) | 26.0 (19.0,34.5) | 24.0 (16.0,32.0) | 23.0 (17.0,32.0) | 19.0 (14.0,28.0) |
| Patient in paid work >20h/week | 2,272 (61.5%) | 241 (67.3%) | 533 (69.4%) | 408 (63.2%) | 193 (63.5%) | 169 (63.8%) | 422 (56.0%) | 302 (55.7%) | n/a |
| Stopped work due to EIA (n, %) | 173 (6.5%) | 11 (4.2%) | 15 (2.5%) | 17 (3.6%) | 24 (10.9%) | 16 (8.7%) | 46 (8.7%) | 41 (10.7%) | n/a |
| Changed job due to EIA (n, %) | 334 (12.6%) | 33 (12.5%) | 56 (9.5%) | 44 (9.4%) | 42 (19.0%) | 19 (10.3%) | 90 (16.9%) | 50 (13.0%) | n/a |
| Reported absenteeism (n, %) | 674 (29.5%) | 75 (31.3%) | 143 (27.3%) | 126 (29.8%) | 59 (31.9%) | 41 (27.2%) | 130 (29.5%) | 100 (31.4%) | n/a |
| Presenteeism | 40.0 (20.0,70.0) | 30.0 (10.0,60.0) | 40.0 (20.0,60.0) | 40.0 (10.0,70.0) | 50.0 (20.0,70.0) | 40.0 (20.0,70.0) | 50.0 (20.0,70.0) | 50.0 (30.0,70.0) | n/a |
| Overall Impairment | 30.0 (1.0,50.0) | 20.0 (1.0,40.0) | 29.4 (9.9,50.0) | 26.3 (1.0,48.4) | 40.0 (20.0,55.6) | 30.0 (10.0,60.0) | 30.0 (10.0,56.9) | 35.5 (1.0,50.2) | n/a |

**Abbreviations**: **IQR** = interquartile range; **HAQ =** Health Assessment Questionnaire; **MSK**-**HQ** = musculoskeletal health questionnaire; **EIA** = early inflammatory arthritis; **n/a** = not applicable.

Median, IQR unless otherwise specified.

**Supplementary Figure S2.** Individual radar plots demonstrating baseline Work Productivity Activity Impairment (WPAI) score by occupation

**Supplementary Figure S3.** Stratified regression by gender examining the association between occupational group and a) having stopped working because of inflammatory arthritis b) absenteeism, c) presenteeism, d) overall work impairment at diagnosis. Reference group is ‘higher managerial, administrative, professional’. Adjusted for age.

1. **Males**


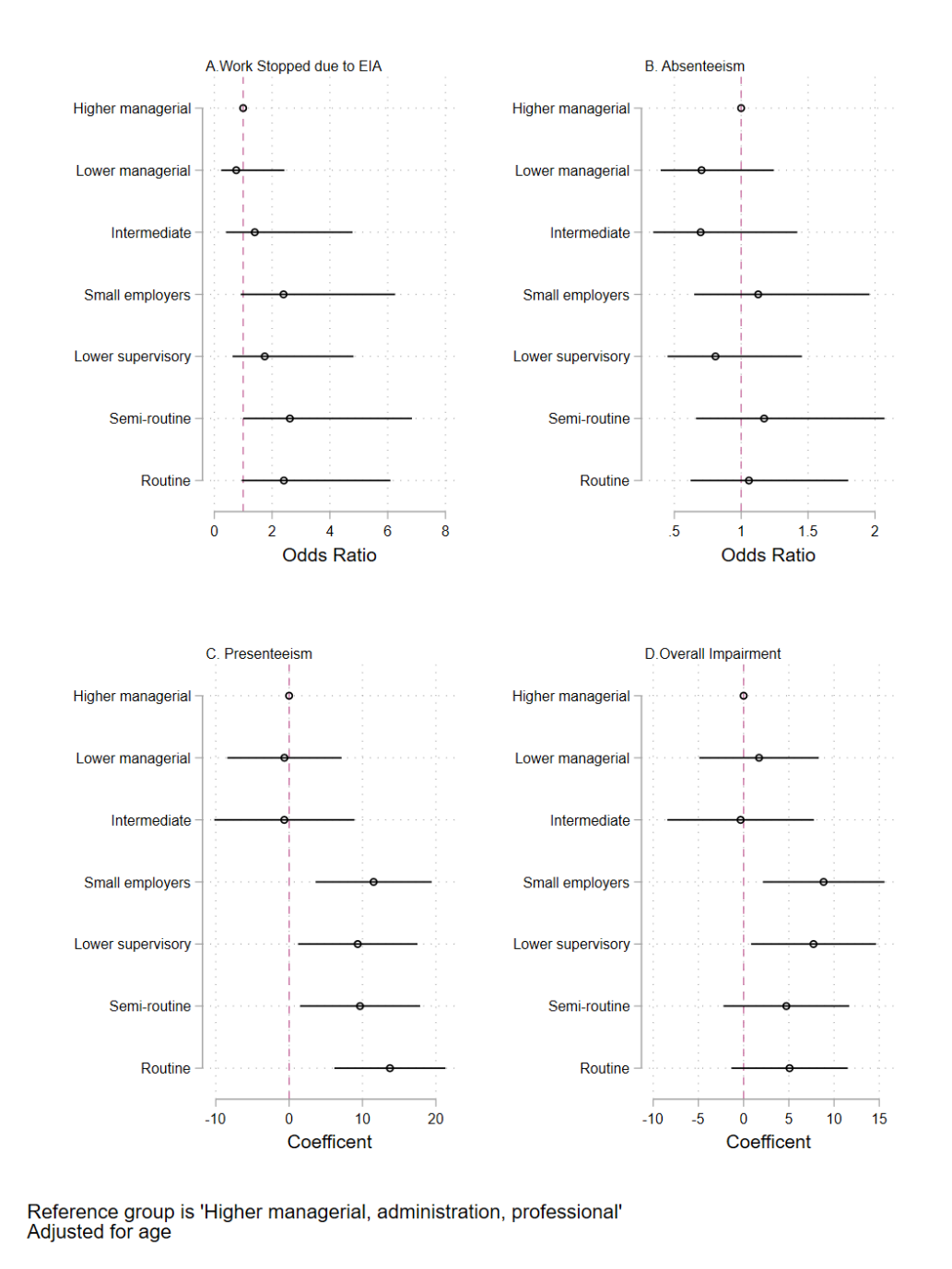


1. **Females**


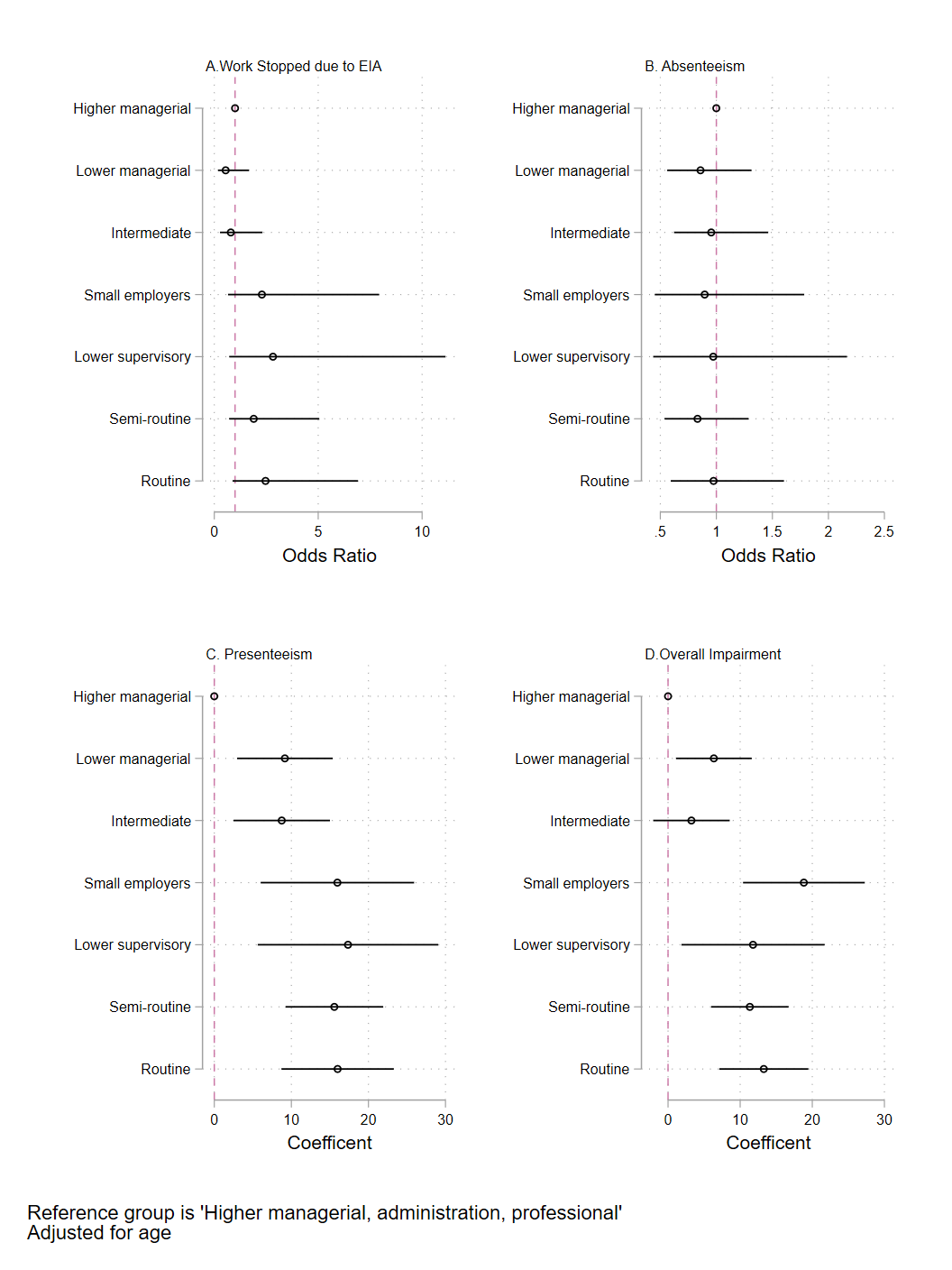

Supplement: kead484_Supplementary_Data [file kead484_supplementary_data.docx]
